# Cardiac glycosides use and the risk and mortality of cancer; systematic review and meta-analysis of observational studies

**DOI:** 10.1371/journal.pone.0178611

**Published:** 2017-06-07

**Authors:** Mohamed Hosny Osman, Eman Farrag, Mai Selim, Mohamed Samy Osman, Arwa Hasanine, Azza Selim

**Affiliations:** Faculty of Medicine, Zagazig University, Zagazig, Egypt; Universidade Federal do Rio de Janeiro, BRAZIL

## Abstract

**Background:**

Cardiac glycosides (CGs) including digitalis, digoxin and digitoxin are used in the treatment of congestive heart failure and atrial fibrillation.

Pre-clinical studies have investigated the anti-neoplastic properties of CGs since 1960s. Epidemiological studies concerning the association between CGs use and cancer risk yielded inconsistent results. We have performed a systematic review and meta-analysis to summarize the effects of CGs on cancer risk and mortality.

**Methods:**

PubMed, Scopus, Cochrane library, Medline and Web of Knowledge were searched for identifying relevant studies. Summary relative risks (RR) and 95% confidence intervals (CI) were calculated using random-effects model.

**Results:**

We included 14 case-control studies and 15 cohort studies published between 1976 and 2016 including 13 cancer types. Twenty-four studies reported the association between CGs and cancer risk and six reported the association between CGs and mortality of cancer patients.

Using CGs was associated with a higher risk of breast cancer (RR = 1.330, 95% CI: 1.247–1.419). Subgroup analysis showed that using CGs increased the risk of ER+ve breast cancer but not ER-ve. Using CGs wasn’t associated with prostate cancer risk (RR = 1.015, 95% CI: 0.868–1.87). However, CGs decreased the risk in long term users and showed a protective role in decreasing the risk of advanced stages. CGs use was associated with increased all-cause mortality (HR = 1.35, 95% CI: 1.248–1.46) but not cancer-specific mortality (HR = 1.075, 95% CI: 0.968–1.194).

**Conclusion:**

The anti-tumor activity of CGs observed in pre-clinical studies requires high concentrations which can’t be normally tolerated in humans. However, the estrogen-like activity of CGs could be responsible for increasing the risk of certain types of tumors.

## Introduction

Cancer represents a major health problem facing the world which is responsible for 13% of all deaths worldwide according to the World Health Organization (WHO) [1]. The global burden of cancer was estimated to be 28.8 million in 2008 [2]. In 2012, 14.1 million new cancer cases were diagnosed with more than 8 million cancer-specific deaths worldwide [3].

In the last century, extensive research has been made to identify the mechanisms of carcinogenicity, however, most cancers still have poor survival [4]. WHO expects an increase in the number of new cancer cases to 22 million and cancer deaths to 13 million annually over the next 2 decades [5]. So, extensive research must be done to identify possible risk factors and potential therapeutic agents.

In terms of risk factors, prevention of cancer requires identification and elimination of cancer-causing agents [6]. In terms of treatment, the exorbitant cost of new drug development is estimated to exceed 1 billion dollars. However, identifying drugs with already established toxicologic, pharmacokinetic and pharmacodynamic profiles which may be effective for unanticipated indications could decrease these costs [7].

Cardiac glycosides (CGs) have been used in the treatment of heart diseases for more than 200 years and were already known to the ancient Egyptians over 3,000 years ago [8]. Cardiac glycosides including digitalis, digoxin and digitoxin are used since the 18th century in the treatment of congestive heart failure and atrial fibrillation [9].

CGs have been investigated as anti-carcinogenic agents since 1960s [10]. Pre-clinical studies have identified several mechanisms for anti-tumor activities of CGs. The main mechanism for CGs is inhibiting Na^+^/K^+^-ATPase activity which increases intracellular Ca^+2^ leading to apoptosis of tumor cells [11]. Other studies found that digitalis activates Src kinase and Cdk5/p25 pathways [12,13]. Digoxin could inhibit the synthesis of (Hypoxia-Inducible Factor-1) HIF-1 alpha protein and the expression of HIF-1 gene which decreased the growth of tumor xenografts [14].

Several epidemiological studies have demonstrated the effect of CGs on the risk of cancer but led to inconsistent results. Multiple reports have suggested that using CGs was associated with a higher risk of estrogen-sensitive tumors such as breast and ovarian cancers [15]. In prostate cancer, studies found that digoxin decreased the risk of prostate cancer [7]. However, other studies reported increased prostate cancer risk in digitoxin users [16]. Also inconsistent results were found in other cancers such as colorectal cancer and male breast cancer.

To date, no systematic review and meta-analysis have been conducted concerning the effect of CGs on cancer risk. Therefore, we provide a comprehensive review of the effect of CGs on cancer risk and mortality of cancer patients.

## Methods

This systematic review was conducted according to: Meta-Analysis of Observational Studies in Epidemiology (MOOSE) and Preferred Reporting Items for Systematic reviews and Meta-Analyses (PRISMA) statement [17,18].

### Literature search

A comprehensive search of literature was carried out using PubMed, Scopus, Cochrane library, Medline and ISI Web of Knowledge using the following terms ("digoxin" or "digitalis" or "digitoxin" or "cardiac glycoside" or “Na^+^ K^+^ ATPase” and "cancer). (S1 Table).

The latest date of search was July 2016 without any search restrictions. The titles and abstracts of identified studies in the search were screened to exclude irrelevant studies. The full texts of relevant articles were assessed carefully to determine whether they were eligible or not. We examined manually the reference lists from relevant articles and literature reviews to further identify potentially relevant studies. All searches were conducted independently by 2 authors and conflicts were solved by discussion.

### Inclusion criteria

A study was included if it fulfilled the following criteria: (a) the study evaluated the exposure of CGs (digoxin, digitalis or digitoxin); (b) the study design was case–control or cohort; (c) the study provided information on the effect of using CGs on cancer risk or mortality of cancer patients; and (d) risk estimates of cancer risk or mortality and 95% confidence intervals (CIs) were reported in the study (or information to calculate them). We included all articles irrespective of publication length; studies published as short reports or conference abstracts, even though the quality assessment of these publications is limited, were included.

### Data extraction

Two independent investigators extracted data from eligible studies. Extracted data included the last name of the first author, year of publication, type of cancer, country of the study, drug of exposure, study design, sample size, number of cancer cases, duration in which cancer cases were diagnosed, risk estimates and corresponding 95% CIs, and covariates adjusted for, in the multivariable analysis. In studies which provided more than one risk estimate, we extracted the one that was adjusted for the largest number of confounding factors. Discrepancies were settled by consensus and discussion amongst reviewers.

### Quality assessment of included studies

Two investigators independently assessed the methodological quality of included studies using the nine-star Newcastle-Ottawa scale (NOS) [19].The assessment was based on eight items, categorized into three main perspectives including selection, comparability, and exposure for case-control studies or outcome for cohort studies. A study was considered high quality if it had a score of 7 or more.

### Statistical methods

Because cancer outcomes were relatively rare, the odds ratios (OR) and hazard ratios (HRs) were considered approximations of relative risks (RRs) [20,21].

In the analysis of mortality, HRs were used because all included studies reported HRs. Summary estimates of RRs and 95% CIs were obtained using the DerSimonian and Laird random-effects model, which considers both within- and between-study variations [22].

If more than one risk estimate was provided in a study that had been stratified by covariates, the estimates were pooled before entering data into the final analysis. We used Rothman/Greenland method to calculate 95% CI if a study reported a standardized incidence ratio without 95% CI [23].

The heterogeneity of our data was assessed by the Q statistic and I^2^ statistic [24]. Heterogeneity among studies was considered significant when P-value was less than 0.1 for the Q statistic or when the I^2^ value was more than 50% [24]. We evaluated publication bias using Egger’s regression test in which P-value less than 0.10 indicated the existence of publication bias [25]. All statistical analyses were performed using Comprehensive Meta-Analysis (CMA) software (version 2.2.064; Biostat, Englewood, NJ; Borenstein et al., 2005) [26].

## Results

**Fig 1:**
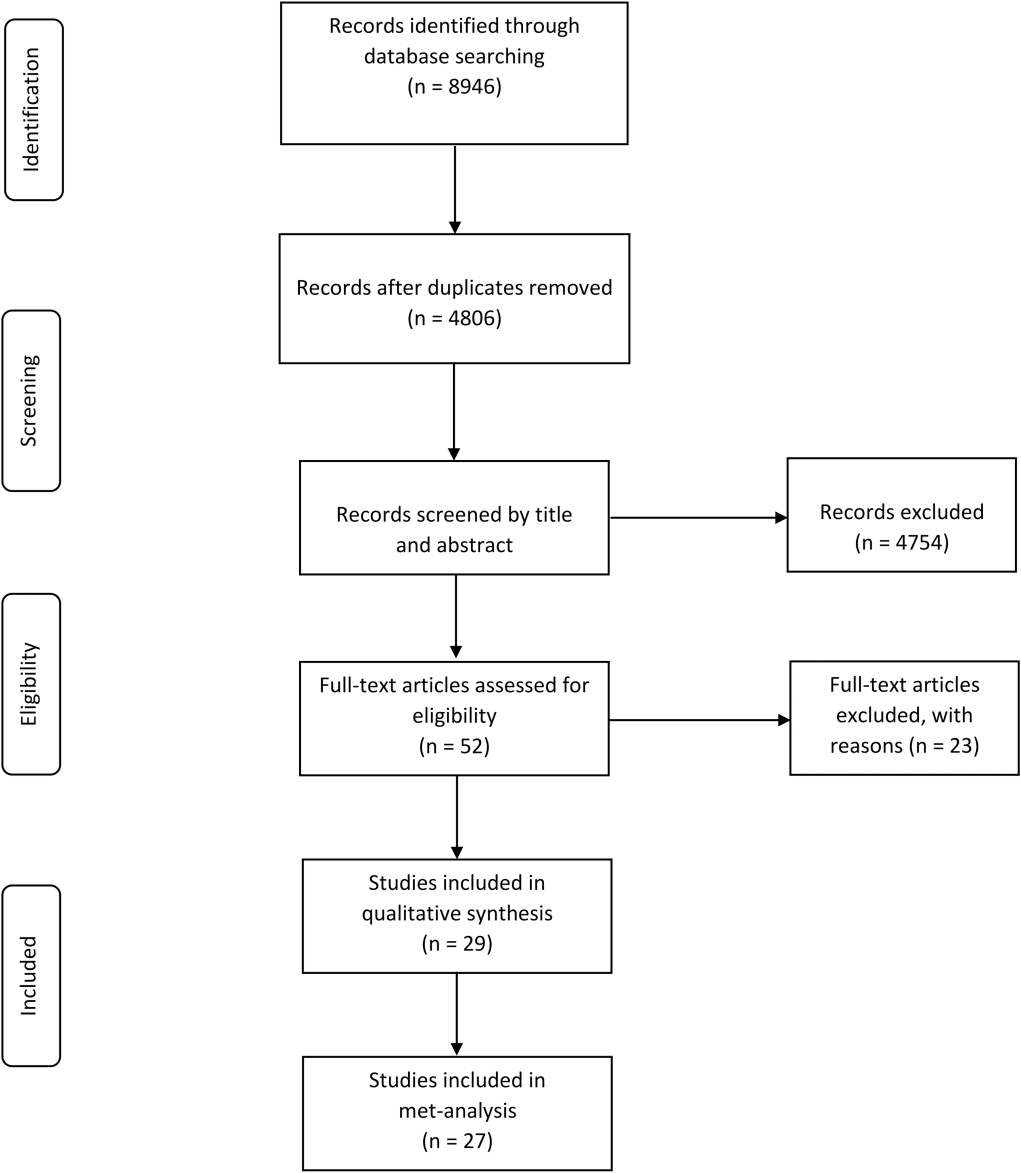
PRISMA chart of the selection of studies included in the qualitative and quantitative syntheses.

The initial search strategy retrieved a total of 8946 citations in all databases (S1 Table). Fig 1 presents the process of study selection.

After removing duplicates, 4806 records were screened by title and abstract.

We identified 52 potentially relevant studies which were assessed in detail with regard to their fulfillment of the inclusion criteria. Twenty-three studies were excluded (S1 File). Five articles were reviews with no original data. The exposure of interest was not CGs use in 7 studies. Six articles were excluded as no usable data were reported. Two studies whose participants were overlapped in other studies were excluded. Four conference abstracts were excluded as 1 reported incomplete data and 3 were published later as journal articles with complete data. Finally, 29 studies concerning the use of CGs and cancer risk and mortality of cancer patients met the eligibility criteria.

### Qualitative synthesis

Fourteen case-control studies [27–40] and Fifteen cohort studies [16,41–54] published between 1976 and 2016 and involving 194,763 cases of 13 types of cancer were included.

Twenty-four studies reported the association between CGs use and cancer risk. Digoxin use was the only drug of exposure in 12 studies, digitalis was the only drug of exposure was in 8 studies and digitoxin was the only drug of exposure in a single study. Two studies included more than one drug and a single study included cardiac glycosides without further specifications. Table 1.

**Table 1:** Characteristics of included studies [CGs use and cancer risk].

Study	Year	Country	Drug	Study Design	Size of Study Sample	# of Cancer Cases	Years of Cancer Diagnosis^1^	RR (95% CI)	Adjustments in Study Analysis
**Risk of Breast Cancer**									
Aromaa	1976	Finland	Digitalis	Case Control	218	109	1973	1.33 (0.73–2.48)	None.
Danielson	1982	United States	Digitalis	Cohort	-	302	1977–1980	1.3 (0.7–2.2) 90% CI	Age.
Friedman	1984	United States	Digitalis	Cohort	143574	20	1969–1980	1.21 (0.78–1.88)	SMR of 10-year age interval.
Haux	2001	Norway	Digitoxin	Cohort	5026	57	1986–1996	1.25 (0.95–1.62)	SIR of year of birth and age.
Ahern	2008	Denmark	Digoxin	Case Control	61251	5,565	1991–2007	1.30 (1.14–1.48)	Age, residence, HRT, anticoagulants, aspirin and NSAIDs.
Biggar	2011	Denmark	Digoxin	Cohort	2,116,029	49,016	1995–2008	1.39 (1.32–1.46)	Attained age and calendar period.
Hartz	2013	United States	Digitalis	Cohort	147,202	-	(1993–1998) + median 8 years follow up	1.46 (1.24–1.72)	Age, race, and the type of study from which data were obtained.
Ahern	2014	United States	Digoxin	Cohort	74,970	4,576	1994–2010	1.40 (1.18–1.65)	Age, height, BMI, age at menarche, age at menopause, alcohol, age at first birth, parity, use of postmenopausal hormones, family history of breast cancer, personal history of benign breast disease, screening mammogram, aspirin, ibuprofen, CLDs and tamoxifen use for breast cancer prevention.
Couraud [34]	2014	United Kingdom	Digoxin	Case Control	9838	898	1988–2012	1.07 (0.90–1.26)	Smoking status, BMI, CG-related indications, HRT and estrogen-based contraceptive drug use, statins, aspirin, oral anticoagulants and antiplatelets, NSAIDs, anti-hypertensive drugs, and anti-diabetic drugs.
**Risk of Prostate Cancer**									
Friedman	1989	United States	Digitalis	Cohort	143574	43	1969–1980	1.48 (1.1–2.0)	SMR of 10-year age interval.
Haux	2001	Norway	Digitoxin	Cohort	4245	108	1986–1996	1.25 (1.03–1.50)	SIR of year of birth and age.
Platz	2011	United States	Digoxin	Cohort	47,884	5002	1986–2006	0.76 (0.60–0.95)	Age, calendar year race, current BMI, BMI at 21, height, family history of prostate cancer, smoking, physical activity, diabetes mellitus, daily caloric intake, linolenic acid, calcium, bacon, fish, tomato sauce use of a vitamin E supplement, use of CLDs, aspirin, ibuprofen, furosemide diuretics, BBs, CCBs, anti-hypertensives, and anti-arrhythmics.
Wright	2014	United States	Digoxin	Case Control	1943	1001	2002–2005	0.58 (0.30–1.10)	Age, race, family history of prostate cancer, PSA screening history. ACEIs, diuretics, statins and aspirin.
Kaapu	2015	Finland	Digoxin	Case Control	49314	24,657	1995–2002	0.96 (0.90–1.02)	Age, use of antihypertensive drugs, CLDs, antidiabetic drugs, NSAIDs, 5a reductase inhibitors and alpha-blockers.
Kaapu	2016	Finland	Digoxin	Cohort	78,615	6,639	1996–2012	1.01(0.87–1.16)	Age, screening trial arm, CLDs, antidiabetic and antihypertensive drugs, aspirin, NSAIDs, 5alpha-reductase inhibitors and alpha-blockers.
**Risk of Colorectal Cancer**									
Friedman	1984	United States	Digitalis	Cohort	143574	35	1969–1980	1.46 (1.05–2.04)	SMR of sex and 10-year age interval.
Friedman	1998	United States	Digitalis	Case Control	4403	1993	1991–1994	1.1 (0.8–1.5)	Age, sex, race, family history of colon cancer, BMI, daily intake of calories, fibers, calcium, physical activity, cigarette smoking and alcohol use.
Haux	2001	Norway	Digitoxin	Cohort	9271	127	1986–1996	1.29 (1.06–1.51)	SIR of year of birth, age and sex.
Boursi	2014	United Kingdom	Digoxin	Case Control	103044	20,990	1995–2013	1.52 (1.40–1.65)	BMI, alcoholism, smoking history, diabetes mellitus, heart disease, chronic NSAIDs use and previous screening colonoscopies
**Risk of Lung Cancer**									
Friedman	1989	United Sates	Digitalis	Cohort	143574	56	1969–1984	1.65 (1.23–2.14)	SMR of sex and 10-year age interval.
Haux	2001	Norway	Digitoxin	Cohort	9271	63	1986–1996	1.35 (1.04–1.74)	SIR of year of birth, age and sex.
Couraud [35]	2014	United Kingdom	Digoxin Lanatoside Digitoxin	Case Control	13557	1237	1988–2012	1.09 (0.94–1.26)	Smoking, BMI indication of CG use alcohol use, history of tobacco-related conditions, history of lung diseases, factors associated with sexual hormonal disorders, use of statins, aspirin, oral anticoagulants and antiplatelet, NSAIDs, diuretics spironolactone, CCBs, ARBs, ACEIs, BBs, oral bisphosphonates, metformin, sulfonylureas, insulins, thiazolidinediones, and amiodarone.
**Risk of Male Breast Cancer**									
Lenfant-Pejovic	1990	France Switzer land	Digitalis	Case Control	346	91	1975–1988	4.1 (1.4–12.4)	None.
Ewertz	2001	Denmark Norway Sweden	Digoxin	Case Control	624	156	1987–1991	1.91(1.05–3.49)	None.
Casagrande	1988	United States	Digitalis Digoxin	Case Control	146	73	1978–1985	0.37(0.11–1.22)	None.
**Risk of Brain Cancer**									
Boursi	2016	United Kingdom	Digoxin	Case Control	5329	1076	1995–2013	Glioblastoma: 0.80 (0.40–1.59)	Obesity, BMI, smoking, diabetes and cardiovascular disease
Seliger	2016	United Kingdom	CG	Case Control	22055	2005	1995–2012	Glioma: 0.47 (0.27–0.81) Glioblastoma: 0.74(0.36–1.55)	BMI, smoking, ACEIs, BBs, diuretics, anti-arrhythmics other than CG, ARBs, CCBs, statins, aspirin, thrombocyte inhibitors, vitamin K antagonists, coronary vasodilators, nitrates, insulin, and oral antidiabetics
						**Risk of Uterine Cancer**			
Biggar	2012	Denmark	Digoxin	Cohort	2,116,029	8,124	1995–2008	1.48, (1.32–1.65)	Age and calendar time.
						**Risk of Ovarian Cancer**			
						7,124	1995–2008	1.06 (0.92–1.22)	
						**Risk of Cervical Cancer**			
						5,001	1995–2008	1.00 (0.79–1.25)	
						**Risk of Leukemia and Lymphoma**			
Haux	2001	Norway	Digitoxin	Cohort	9271	53	1986–1996	1.41 (1.06–1.85)	SIR of year of birth, age and sex.
						**Risk of Kidney and Urinary Cancers**			
						59	1986–1996	1.14 (0.87–1.47)	
						**Risk of Melanoma and Skin Cancers**			
						61	1986–1996	1.23 (0.94–1.58)	
**Risk of Uterine Cancer Non-Hodgkin’s Lymphoma**									
Bernstein	1992	United States	Digitalis	Case Control	1238	619	1979–1982	1.55 (0.99–2.43)	None.

^1^Duration in which cancer cases were diagnosed.

*Abbreviations: (ACEIs) Angiotensin-Converting-Enzyme Inhibitors, (ARBs) Angiotensin Receptor Blockers, (BBs) Beta Blockers, (BMI) Body Mass Index, (CCBs) Calcium Channel Blockers, (CG) Cardiac Glycosides, (CLDs) Cholesterol-Lowering Drugs, (HRT) Hormone Replacement Therapy, (NSAIDs) Non-Steroidal Anti-Inflammatory Drugs, (PSA) Prostate-Specific Antigen, (SIR) Standardized Incidence Ratio, (SMR) Standardized Morbidity Ratio.

Six studies reported the association between digoxin use and mortality of cancer patients. Digoxin use was the drug of exposure in these 6 studies. Table 2.

**Table 2:** Characteristics of included studies [digoxin use and mortality].

Study	Year	Type of Cancer	Country	Drug	Study Design (Follow up Period)	# of Cancer Cases	Number of Cancer Specific Deaths	HR (95% CI)	Adjustments in Study Analysis
Flahavan	2014	**Prostate Cancer**	Ireland	Digoxin	Cohort (Median 4.3 years)	5732	1098	Adjusted HR for Cancer Specific Mortality 1.13 (0.91–1.42) All-cause mortality 1.24 (1.07–1.43)	Age, comorbidity score, tumor stage, grade, smoking status at diagnosis, year of incidence, warfarin exposure and statin exposure.
Karasneh [52]	2015	**Colorectal Cancer**	United Kingdom	Digoxin	Cohort (Mean 4.8 years)	10,357	2,724	Adjusted HR for Cancer Specific Mortality 1.11 (0.91–1.36) All-cause mortality 1.52 (1.34–1.73)	Year of diagnosis, age at diagnosis, gender, within 6 months (surgery, radiotherapy, chemotherapy), site (colon or rectum), comorbidities prior to diagnosis, low-dose aspirin, statins, metformin, and ACEIs
Karasneh [51]	2015	**Breast Cancer**	United Kingdom	Digoxin	Cohort (Mean 5.2 years)	17,842	2219	Adjusted HR for Cancer Specific Mortality 0.91 (0.72–1.14) All-cause mortality 1.24 (1.08–1.41)	Year of diagnosis, age at diagnosis, within 6 months (surgery, radiotherapy, chemotherapy hormone therapy), HRT prior to diagnosis, comorbidities prior to diagnosis, low-dose aspirin, statins, and spironolactone
Boursi	2016	**Glioblastoma**	United Kingdom	Digoxin	Cohort	1076	-	All-cause mortality 1.50 (0.74–3.04)	Age, sex, duration of follow-up before cancer diagnosis, obesity (BMI > 30), smoking, diabetes and cardiovascular disease.
Karasneh	2016	**Prostate Cancer**	United Kingdom	Digoxin	Cohort (Mean 5 years)	13,134	2010	All-cause mortality 1.39 (1.23–1.56) Cancer-Specific Mortality 1.13 (0.93–1.37)	Year of diagnosis, age at diagnosis, within 6 months (surgery, radiotherapy, chemotherapy, androgen deprivation therapy, estrogen therapy), comorbidities prior to diagnosis aspirin, statins, metformin, ACEIs spironolactone and deprivation (in fifth)
Vogel	2016	**Epithelial Ovarian Cancer**	United States	Digoxin	Cohort	762	-	All-cause mortality 1.29 (0.81–2.06)	Age, heart disease and Charlson comorbidity score

*Abbreviations: (ACEIs) Angiotensin-Converting-Enzyme Inhibitors, (BMI) Body Mass Index, (HRT) Hormone Replacement Therapy.

The Newcastle-Ottawa Scale results showed that the average overall score was 7.4 (range 5–9) suggesting a good methodological quality of included studies (S2 Table).

### Quantitative synthesis

#### Risk of cancers

We conducted a meta-analysis to detect the association between CGs exposure and the risk of each cancer separately (breast, prostate, colorectal, lung, male breast, and glioblastoma). Nine studies presented a risk estimate for breast cancer, 6 for prostate cancer, 4 for colorectal cancer, 3 for lung cancer, 3 for male breast cancer and 2 for glioblastoma. No meta-analysis was conducted for cancers (ovary, uterus, cervix, non-Hodgkin’s’ lymphoma, leukemia, kidney, urinary, melanoma, and skin) because only a single study was available for each type. The risk of these cancers is reported separately in Table 1.

#### Breast cancer

**Fig 2:**
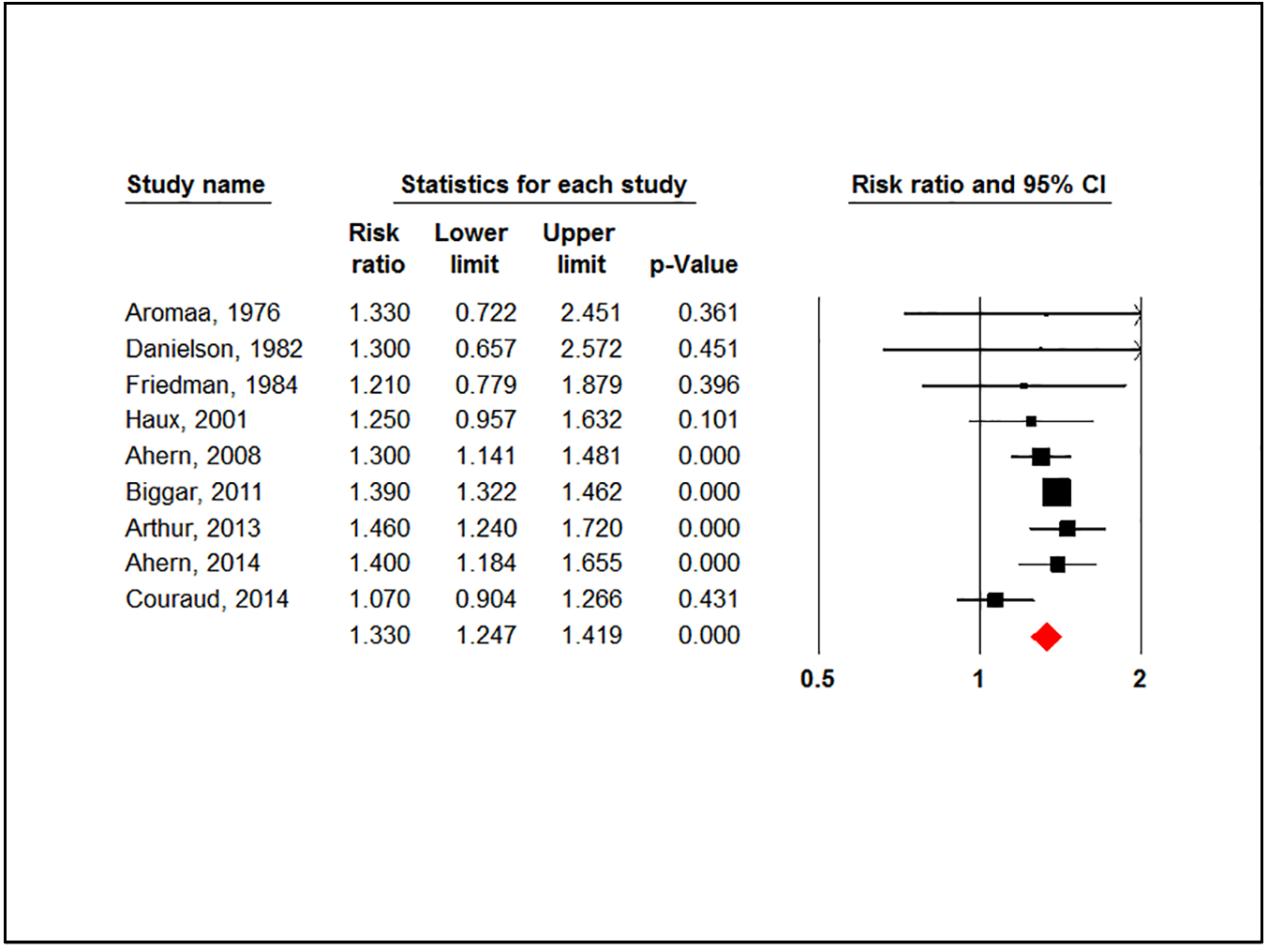
Forest plot for association between cardiac glycosides use and the overall risk of breast cancer.

Using CGs was associated with a significant higher risk of breast cancer (RR = 1.330, 95% CI: 1.247–1.419, P-value = 0.000). Fig 2.

There was no evidence of heterogeneity (P-value = 0.232, I^2^ = 23.78%) or publication bias (P-value = 0.271). After stratification by study design, the risk in case-control studies was (RR = 1.2, 95% CI: 1.031–1.39, P-value = 0.019) and (RR = 1.389, 95% CI: 1.328–1.454, P-value = 0.000) in cohort studies. (-S2 File).

The overall risk didn’t change when we stratified analysis by the drug of exposure (RR = 1.331, 95% CI: 1.227–1.443, P-value = 0.000). Digitalis was associated with the highest risk (RR = 1.415, 95% CI: 1.415, P-value = 0.00) compared to digoxin (RR = 1.301, 95% CI: 1.171–1.445, P-value = 0.00). (S2 File).

Moreover, in a subgroup analysis based on the duration of drug use, patients who used digoxin for 3 years or more had also a higher risk of breast cancer (RR = 1.279, 95% CI: 1.098–1.490, P-value = 0.002). (S2 File).

In a stratified analysis by estrogen receptor (ER) status, a higher risk of ER+ve breast cancer was found in digoxin users (RR = 1.332, 95% CI: 1.249–1.421, P-value = 0.000). However, no such association was found in ER-ve tumors (RR = 0.984, 95% CI: 0.611–1.584, P-value = 0.946). (S2 File).

#### Prostate cancer

**Fig 3:**
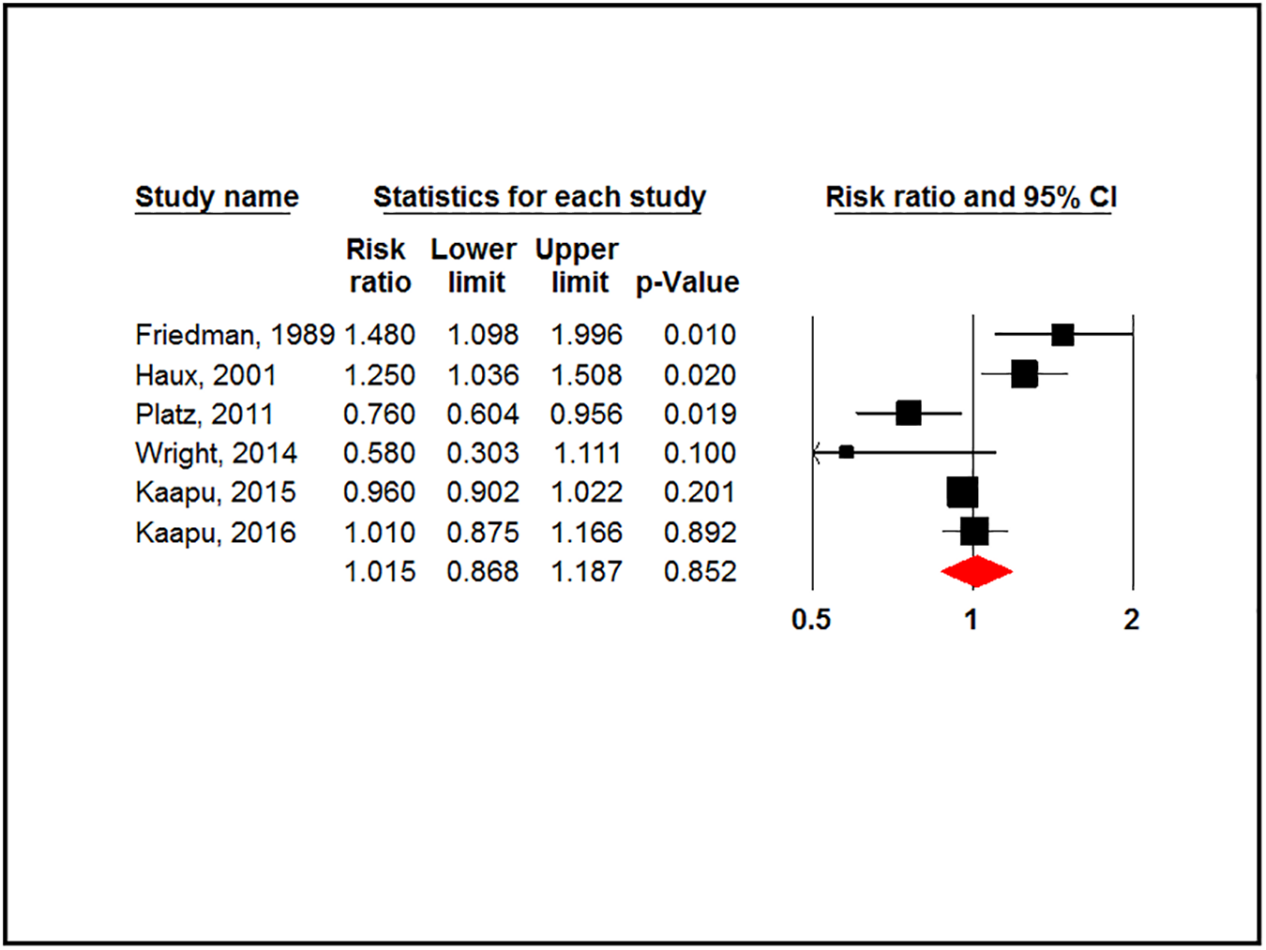
Forest plot for association between cardiac glycosides use and risk of prostate cancer.

There was no association between CGs and prostate cancer (RR = 1.015, 95% CI: 0.868–1.87, P-value = 0.852). Fig 3.

However, a significant level of heterogeneity was detected (P-value = 0.001, I^2^ = 76.756%). The Egger test showed no evidence of publication bias (P-value = 0.797).

Stratification by study design showed no significant association in cohort studies (RR = 1.08, 95% CI: 0.851–1.372, P-value = 0.526) nor in case-control studies (RR = 0.831, 95% CI: 0.532–1.299, P-value = 0.417). High evidence of heterogeneity was observed in cohort studies compared to case-control studies (P-value = 0.001 and 0.13 respectively). (S3 File).

Digoxin users had a lower risk of prostate cancer when they used digoxin for 3 years or more and 5 years or more, respectively. (RR = 0.910, 95% CI: 0.822–1.008, P-value = 0.070) (RR = 0.893, 95% CI: 0.785–1.016, P-value = 0.086). There was no evidence of heterogeneity (P-value > 0.1). (S3 File).

Stratification of analysis by cancer stage and Gleason score showed that digoxin users had a non-significant lower risk of advanced prostate cancer (≥T3b, N+ or M+ at diagnosis) (RR = 0.880, 95% CI: 0.765–1.012, P-value = 0.074). Digoxin decreased the risk of prostate cancer with Gleason score 7 or more (RR = 0.804, 95% CI: 0.676–0.956, P-value = 0.014). (S3 File).

#### Colorectal cancer

Using CGs was associated with a significant higher risk of colorectal cancer (RR = 1.38, 95% CI: 1.203–1.582, P-value = 0.000) with no significant heterogeneity (P-value = 0.116, I^2^ = 49.214%) or publication bias (P-value = 0.227).

After stratification by study design, the risk wasn’t significant in case-control studies (RR = 1.342, 95% CI: 0.986–1.827, P-value = 0.062). However, the risk remained significant in cohort studies (RR = 1.326, 95% CI: 1.134–1.550, P-value = 0.00). There was a significant evidence of heterogeneity in case-control studies compared to cohort studies (P-value = 0.051 and 0.519 respectively). (S4 File).

#### Other cancers

CGs users had a higher risk of lung cancer (RR = 1.315, 95% CI: 1.025–1.687, P-value = 0.031). They also had a higher risk for male breast cancer but wasn’t statistically significant (RR = 1.501, 95% CI: 0.481–4.686, P-value = 0.485). In both analyses, there was a significant level of heterogeneity (P-value < 0.1) but without any evidence of publication bias (P-value > 0.05). (S4 File).

CGs were associated with a lower risk of glioblastoma but wasn’t statistically significant (RR = 0.771, 95% CI: 0.467–1.237, P-value = 0.31) with no evidence of heterogeneity between studies (P-value = 0.88). (S4 File).

#### Mortality of cancer patients

We conducted a meta-analysis to detect the association between using CGs and mortality in all cancers. Due to the little number of available studies, we couldn’t analyze the risk in each type of cancer alone. Six studies provided a risk estimate for all-cause mortality and 4 of them reported also cancer-specific mortality. The drug of exposure was digoxin in all included studies.

**Fig 4:**
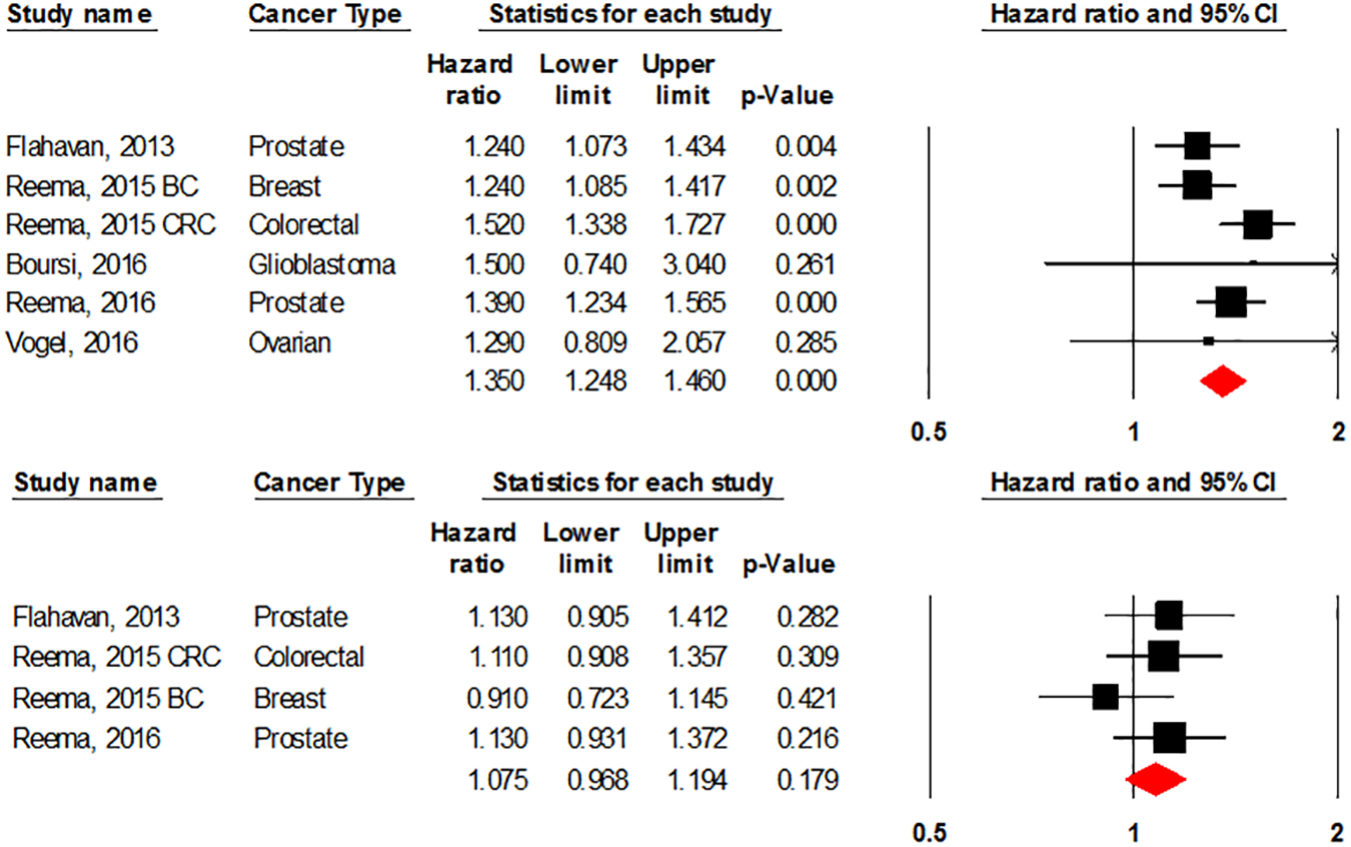
Forest plot for association between cardiac glycosides use and all-cause mortality (above) and cancer specific mortality (below). BC: Breast Cancer, CRC: Colorectal Cancer.

Digoxin was associated with increased all-cause mortality in cancer patients (HR = 1.35, 95% CI: 1.248–1.46, P-value = 0.00). However, no association was found between using digoxin and cancer-specific mortality (HR = 1.075, 95% CI: 0.968–1.194, P-value = 0.179). Fig 4.

There was no evidence of heterogeneity between studies in all-cause mortality analysis (P-value = 0.257, I^2^ = 23.584%) and cancer-specific mortality analysis (P-value = 0.463, I^2^ = 0.00%). We found no evidence of publication bias in both analyses (P-value = 0.926 and 0.313) respectively.

## Discussion

Despite laboratory evidence of anti-proliferative effects of CGs on tumor cells, our results did not provide any beneficial clinical effects in lowering cancer risk or improving cancer survival.

### Breast cancer

Our meta-analysis showed that using CGs was associated with 33% higher risk of breast cancer. Subgroup analysis by ER status showed the same 33% increased risk in ER+ve tumors despite a non-significant association between CGs and ER-ve tumors. CGs are known to have estrogen-mimetic activities as the basic structure of digitalis compounds is similar to that of estradiol [15]. CGs also share a common molecular structure that includes a steroid nucleus [55]. The ability of CGs to bind to estrogen receptors could be responsible for the higher risk of estrogen-sensitive tumors as breast cancer in CGs users [56,57]. Digoxin and digitoxin were able to interact with the estrogen receptors in rat uteri and digitoxin could inhibit the estradiol to its binding site[58]. In a receptor assay study, digitoxin could interact with the estrogen receptor in human pre-menopausa uterine cytoso but couldn’t interact with the steroid binding protein in plasma suggesting that the estrogenic activity of CGs is attributed to receptor interactions not plasma protein alterations [59]. Digitoxin was also able to act as estrogen in oophorectomized adrenalectomized rats [59].

The role of estrogen in promoting breast cancer has been extensively studied. Estradiol was able to induce neoplastic transformation of human breast cells in mouse models [60]. Many studies have documented that menopausal hormone therapy increases the incidence of breast cancer.[15] Several preclinical studies have investigated CGs as anti-cancer agents. Digoxin was able to inhibit topoisomerase II catalytic activity at a concentration of (100 nM) [61]. Also, it mediated apoptosis in the estrogen-independent MDA-MB-231 breast cancer cells [62]. Digitoxin was able to activate EGR1 which enhanced the expression of apoptotic genes and decrease the expression of the CDC16 gene [63].

A study showed that digoxin and digitoxin were able to inhibit breast cancer proliferation and activate extracellular signal-regulated kinase (ERK1/2) at concentrations lower than 100 nM in breast cancer cell lines [12]. However, the therapeutic plasma levels of digoxin and digitoxin are considered to be in the ranges of 0.6 to 1.9 nM and 13 to 33 nM, respectively [64]. This could explain why these in-vitro anti-carcinogenic effects of CGs on breast cancer can’t be observed clinically.

When we stratified analysis by the drug of exposure, the risk of breast cancer increased in digitalis users to 42% compared to 30% in digoxin and 25% in digitoxin. These variations in the risk could be due to differences between studies as most studies of digitalis were conducted in the previous century with little or no adjustments for confounders and only one study on digitoxin was available. Another explanation would be the different potencies of CGs compounds in tumor inhibition. In-vitro studies have reported a superior activity of digitoxin over digoxin on inhibiting the growth of breast cancer cells [12,65].

### Prostate cancer

No clear association was found between using CGs and overall prostate cancer risk (RR = 1.02) but a significant level of heterogeneity between studies was observed (I^2^ = 76.76%). When we stratified analysis by study design, no significant association between CGs and prostate cancer risk was found either in cohort or case-control studies. However, heterogeneity was significant only in cohort studies. When we excluded studies which had no adjustments for covariates (Friedman [42] and Haux [16]), no significant change was found in overall cancer risk (RR = 0.916, 95% CI: 0.806–1.040, P-value = 0.175). But the level of heterogeneity decreased to (I^2^ = 54.84) suggesting that significant heterogeneity could be attributed to un-adjustments of covariates in included studies.

Longer duration of using CGs showed a protective role of digoxin against prostate cancer risk but wasn’t statistically significant (RR = 0.91 for 3 years or more of drug use and RR = 0.89 for 5 years or more). Only a single study reported the risk of prostate cancer in patients who used digoxin 10 years or more (RR = 0.54, 95% CI: 0.37–0.79, P-value<0.001). This supports the idea of the protective effects of CGs in long term users [66].

CGs users had a 20% reduction in the risk of prostate cancer with Gleason score ≥ 7 (RR = 0.80) and a non-significant lower risk of advanced prostate cancer (RR = 0.88). Because 75% of metastatic prostate cancers are hormone sensitive, estrogen has been used as a method of androgen blockade in the treatment of advanced prostate cancer [67].

Carruba et al, demonstrated that estradiol could significantly inhibit the growth of prostate cancer cells [68]. Diethylstilbestrol (DES) was found to have direct cytotoxic effects against various androgen-independent prostate cancer cell lines [69]. Administration of 17-β estradiol suppressed castration-resistant prostate cancer growth which decreased mortality in multiple castration-resistant xenograft models in-vivo [70]. Clinical trials revealed that parenteral estrogen was as effective as combined androgen deprivation in the treatment of metastatic prostate cancer [71]. These observations suggest that the estrogenic activity of CGs is responsible for decreasing the risk of advanced stages of prostate cancer.

Although our results didn’t confirm a reduction in the risk of prostate cancer in CGs users, digoxin showed a protective effect in long term users and in advanced cancer stages. In-vitro studies showed that CGs were able to induce apoptosis in androgen-dependent and independent prostate cancer cells and also in metastatic prostate cancer cells [72,73].

In another study, digitalis decreased the secretion of prostate-specific antigen (PSA) by downregulating prostate-derived Ets factor [74].

The mechanism by which CGs can alter the proliferation of prostate cancer cells remains unclear. Zavareh et al, demonstrated that CGs were able to inhibit tumor cell migration and invasion through blocking N-glycosylation–mediated processes [75].

A pre-clinical study showed that digoxin increases intracellular Ca^+2^ causing changes in the activity of cyclin-dependent kinase Cdk5, cleavage of p35 and formation of p25 leading to prostate cancer cells apoptosis [76].

Zhang H et al, found that digoxin was able to inhibit hypoxia-inducible factor 1 (HIF-1) which regulates the expression of vascular endothelial growth factor (VEGF). Inhibiting HIF-1 can inhibit angiogenesis and block the growth of prostate tumors [14].

### Colorectal cancer

Our meta-analysis showed that CGs users had a higher risk of colorectal cancer (RR = 1.38). Although previous pre-clinical studies found that CGs were able to down regulate α1 Na^+^/K^+^-ATPase which increases Src activity and promotes cell proliferation, migration and invasion in colorectal cancer cell lines [77]. Despite the estrogen-mimetic activity of digoxin, a previous study showed that risk of colorectal cancer is decreased with estrogen hormonal replacement therapy [78]. This rules out the estrogenic pathway mechanism of CGs from increasing colorectal cancer risk. So, the mechanism underlying increased colorectal cancer in CGs users remains unclear. so, further research is needed to explore the biological pathways of this relationship. Only a single study reported the association between the duration of digoxin exposure and risk of colorectal cancer. The risk of colorectal cancer was higher in digoxin users <5 years and 5–10 years but wasn’t statically significant (RR = 1.06 and RR = 1.21) respectively. However, long-term users (more than 10 years) had non-significant lower risk of colorectal cancer (RR = 0.73, 95% CI: 0.41–1.30, P-value = 0.29) [33].

### Lung cancer

Using CGs was associated with a higher risk of lung cancer (RR = 1.32). We found a high evidence of heterogeneity (I^2^ = 73.1) which was attributed to few available studies and lack of adjustments for important confounders including smoking and alcohol. The only study which adjusted for potential confounders didn’t confirm this association (RR = 1.09, 95% CI 0.94–1.26) [35].

The higher risk of lung cancer can be attributed to the estrogen-mimetic properties of digoxin. There is a higher incidence of lung cancer among females than males which suggests the involvement of sexual hormones in lung carcinogenesis. In a published meta-analysis, hormone replacement therapy was associated with a higher risk of lung cancer (OR = 1.76, 95% CI: 1.072–2.898) [79].

Immunohistochemical studies found a significant higher expression of estrogen receptors beta (ER β) in lung cancer lines compared to normal lungs [80]. In lung cancer cell lines, 17β-estradiol increased cell growth in vitro and in tumor xenografts [81]. In a lung cancer mouse model, administration of β-estradiol at physiological levels doubled the number of tumors and promoted tumor progression [82].

These pre-clinical studies suggest a role of estrogen in lung carcinogenesis and thus support the role of CGs in increasing lung cancer risk.

However, in our analysis, a single study only was adjusted for smoking which is considered a risk factor for both lung cancer and cardiovascular diseases. This study found a non-significant association between lung cancer and digoxin [35]. However, the other two studies which had no adjustment for smoking found a significant association between using CGs and lung cancer. We couldn’t reach a definitive conclusion due to lack of smoking adjustments in the other two studies. Further studies adjusted for smoking and other potential confounders are needed to reach a definitive conclusion.

### Male breast cancer

Three case-control studies reported the association between using CGs and the risk of male breast cancer. Using CGs was associated with a non-significant higher risk of male breast cancer (RR = 1.50). A high evidence of heterogeneity was observed (I^2^ = 78.1). This heterogeneity existed because studies had small sample size without inadequate ascertainment of exposure, used other types of cancer as a control group and didn’t adjust the analysis for potential confounders. These limitations existed because male breast cancer is rare and the studies are relatively old.

Gynecomastia is reported to be a side effect of digoxin which is attributed to its estrogen-like action [83]. A case-control study of 74 cases of male breast cancer found a significant increase in the risk of male breast cancer associated with gynecomastia (OR:23.42, 95% CI: 4.65–117.97) [84].

Estrogen receptors were found to be highly expressed in male breast cancer tissues which suggest that male breast cancer is a hormone-dependent tumor [85]. High levels of estrogen were found to increase the risk of male breast cancer. Chemical castration by prolonged administration of high doses of estrogen was associated with higher risk of male breast cancer [86].

In a report by Kanhai et al, men chemically castrated for prostate cancer had acinar and lobular changes in their breast tissue, which could increase the risk of developing breast cancer [87]. Gynecomastia induced by CGs and their estrogen-like properties could suggest a role in increasing male breast cancer risk. However, well-designed epidemiological studies are needed to confirm this relationship.

### Brain cancers

Although in-vitro studies found digitoxin to be able to sensitize glioma to tumor necrosis factor-related apoptosis, our analysis found no association between using CGs and the risk of glioblastoma (RR = 0.771, 95% CI: 0.467–1.237). When Seliger et al stratified analysis by the duration of drug use, the risk of glioma was (OR = 0.57, 95% CI: 0.29–1.14, P-value = 0.111) in long-term users (≥ 25 months) and (OR = 0.35, 95% CI 0.14–0.88, P-value = 0.025) in short-term users of CGs (< 25 months) [39].

In a case-control study, long-term use of oral contraception was inversely associated with glioma [88]. Thus, the estrogen-like action of CGs could be responsible for this substantial decrease in the risk of glioblastoma. A recent study showed a detectable expression of ERβ in glioma cells. The proliferation of glioma cells was inhibited when they were treated by ERβ agonists which reduced also in-vivo tumor growth in a xenograft model [89]. This suggests a role of digoxin in decreasing the risk of glioma. Only two case-control studies were available for analysis which could explain the non-significant association. So, more studies are needed to investigate this relationship.

### Gynecological cancers

Biggar et al found no increase in the risk of ovarian or cervical cancers in digoxin exposed women. However, the risk increased significantly for uterus cancers [46]. The risk of uterus cancers increases with using hormone replacement therapy, so the estrogenic activity of CGs could be responsible for increasing this risk. [90]. Biggar et al, referred the non-significant association between ovarian and cervical cancers to the hormonal insensitivity of these cancers. They supported their idea by the conflicting results of epidemiological studies about the role of estrogen in ovarian and cervical cancers. In our opinion, these controversial results occurred due to neglecting the role of estrogen receptors expression in cancer cells. Immunohistochemical studies found that only 33% of ovarian cancer patients expressed estrogen receptors [91,92]. In a study of cervical cancer, 66.7% of cases were ER+ve [93].

These immunohistochemical studies explain that 2/3 of ovarian cancer patients and 1/3 of cervical cancer patients have no estrogen receptors. In a pre-clinical study, the estrogen couldn’t promote either dysplasia or cervical cancer in ERα-ve mouse model [94]. Continuous exposure to exogenous estrogen contributed to the progression of ERα+ve cervical cancer in a mouse model [95]. In another mouse model, estrogen receptor antagonists effectively prevented and treated ERα+ve cervical cancer [96].

These studies suggest a role of estrogen in ovarian and cervical cancers but only in estrogen-positive tumors. Since epidemiological studies didn’t take into account these molecular differences, we can’t rely on data that found no association between using CGs and the risk of ovarian and cervical cancers. So, studies that take into account the estrogen receptor expression in gynecological cancers are needed to study these associations.

### Lymphoproliferative cancers

Bernstein et al, reported a higher risk of non-Hodgkin’s lymphoma associated with using digitalis (OR = 1.55, 95% CI: 0.99–2.43) [29]. The risk was much higher in females than males (OR = 2.4, 95% CI:1.31–4.38 and OR = 0.75, 95% CI: 0.36–1.59) respectively. This suggested a role of hormonal factors to influence the risk of lymphoma. Haux et al, found that the risk of leukemia and lymphoma was the highest among all cancers in digitoxin users [16].

Epidemiological studies showed conflicting results regarding the association between lymphoma and sexual hormones. A cohort study found that hormone replacement therapy users had a higher risk for nodal follicular non-Hodgkin’s lymphoma (RR = 3.3, 95% CI: 1.6–6.9) However, no significant risk for diffuse or chronic was observed [97]. A case-control study showed that using menopausal hormone therapy was associated with a lower risk of all subtypes of non-Hodgkin’s lymphoma (OR = 0.68, 95% CI: 0.48, 0.98). Using also oral contraception reduced the risk of non-Hodgkin’s lymphoma (OR = 0.68, 95% CI: 0.49, 0.94) [98].

Another observational study observed a statistically significant 29% higher risk of non-Hodgkin’s lymphoma in estrogen users (HR = 2.25, 95% CI: 1.17–4.33) [99]. These contradicting results could be due to different properties of every subtype of lymphoma. So, studying the effect of estrogen and estrogen-like substances on every subtype of lymphoma alone is essential to understand these relationships.

### Mortality

In terms of all-cause mortality, using digoxin was significantly associated with a higher rate of deaths (HR = 1.35). This was expected due to higher levels of cardiovascular comorbidity in the digoxin-exposed groups [49,50]. This association couldn’t be found in cancer-specific mortality (HR = 1.075, 95% CI: 0.968–1.194). This result does not support the findings of preclinical studies which suggested that the inhibitory effects of CGs on cancer cells can improve survival in cancer patients.

### Limitations

The potential limitations of our study should be considered when interpreting these results. Not all studies were adjusted for potential confounders which could affect our results. Only a limited number of studies was available for some types of cancers. Significant level of heterogeneity was detected in the analysis of the risk of prostate, lung, and male breast cancers. A limited number of studies was available for subgroup analyses of ER status in breast cancer and Gleason score in prostate cancer. Most studies collected data about drug exposure retrospectively.

To the best of our knowledge, this is the first systematic review and meta-analysis to provide a comprehensive and precise estimate of the role of CGs in the risk and survival of cancer patients. No dose-response analysis was needed because CGs dosage is usually uniform, which minimizes dose-response effects [44].

Although pre-clinical studies found that CGs can be potent anti-neoplastic agents due to several mechanisms including Na^+^/K^+^-ATPase inhibition and HIF-1α inhibition, our meta-analysis couldn’t confirm these results. In pre-clinical studies, digoxin has been shown to inhibit HIF-1α in prostate cancer cell lines at concentrations of 100 nM and prostate cancer cell proliferation at 23–255 nM. However, these concentrations are considerably higher than the therapeutic plasma concentrations normally tolerated in humans, 1.6 ± 1.0 nM. This could explain why typical levels of digoxin in humans can’t exhibit these anti-neoplastic properties [49].

To benefit from the potent cytotoxicity of CGs, derivatives have been developed for cancer treatment. Anvirzel™ one of these derivatives can inhibit the catalytic activity of the Na^+^/K^+^-ATPase pump and fibroblast growth factor-2 (FGF-2) in a concentration and time-dependent manner [10]. Anvirzel was assessed in phase 1 clinical trial to determine the maximum tolerated dose (MTD) and safety [100].

The estrogen-mimetic activity of CGs may be responsible for increasing the risk of estrogen-sensitive tumors. CGs users have a higher risk of breast tumors, especially ER+ve. Using CGs isn’t associated with prostate cancer risk, however, prolonged use could decrease the risk especially in the advanced stages. CGs were associated with a higher risk of all-cause mortality. However, no association was found between using CGs and cancer-specific mortality.

## Supporting information

S1 Table Literature search results. (DOCX)

S2 Table Methodological quality of the included studies, based on the NOS for assessing the quality of epidemiological studies. (DOCX)

S1 File Excluded studies with reasons. (DOCX)

S2 File Forest plots of breast cancer analysis. (DOCX)

S3 File Forest plots of prostate cancer analysis. (DOCX)

S4 File Forest plots of cancers colorectal, glioblastoma, male breast and lung. (DOCX)
