# Supplementary material for: Cardiac glycosides use and the risk and mortality of cancer; systematic review and meta-analysis of observational studies
Source: PLoS One. 2017 Jun 7;12(6):e0178611. doi: 10.1371/journal.pone.0178611 (PMC5462396; doi:10.1371/journal.pone.0178611)
Supplement: S1 Table — (DOCX) [file pone.0178611.s001.docx]

Supplementary Table 1. Literature Search Results

| Database | Search Words | Results |
| --- | --- | --- |
| PubMed through NLM | (Digoxin [All Text] AND Cancer [All Text]) | 555 |
|  | (Digitalis [All Text] AND Cancer [All Text]) | 193 |
|  | (Digitoxin [All Text] AND Cancer [All Text]) | 102 |
|  | (“Cardiac Glycoside” [All Text] AND Cancer [All Text]) | 1708 |
|  | (“Na+ K+ ATPase” [All Text] AND Cancer [All Text]) | 960 |
| Total | | 3518 |
|  | | |
| Cochrane Library | (Digoxin [All Text] AND Cancer [All Text]) | 65 |
|  | (Digitalis [All Text] AND Cancer [All Text]) | 21 |
|  | (Digitoxin [All Text] AND Cancer [All Text]) | 0 |
|  | (“Cardiac Glycoside” [All Text] AND Cancer [All Text]) | 1 |
|  | (“Na+ K+ ATPase” [All Text] AND Cancer [All Text]) | 1 |
| Total | | 88 |
|  | | |
| ISI web of knowledge | (Digoxin [All Text] AND Cancer [All Text]) | 340 |
|  | (Digitalis [All Text] AND Cancer [All Text]) | 117 |
|  | (Digitoxin [All Text] AND Cancer [All Text]) | 101 |
|  | (“Cardiac Glycoside” [All Text] AND Cancer [All Text]) | 104 |
|  | (“Na+ K+ ATPase” [All Text] AND Cancer [All Text]) | 460 |
| Total | | 1122 |
|  | | |
| Medline Via EBSCO | (Digoxin [All Text] AND Cancer [All Text]) | 401 |
|  | (Digitalis [All Text] AND Cancer [All Text]) | 174 |
|  | (Digitoxin [All Text] AND Cancer [All Text]) | 90 |
|  | (“Cardiac Glycoside” [All Text] AND Cancer [All Text]) | 93 |
|  | (“Na+ K+ ATPase” [All Text] AND Cancer [All Text]) | 580 |
| Total | | 1338 |
|  | | |
| Scopus | (Digoxin AND Cancer [Title, Abstract & Keywords]) | 1486 |
|  | (Digitalis AND Cancer [Title, Abstract & Keywords]) | 340 |
|  | (Digitoxin AND Cancer [Title, Abstract & Keywords]) | 190 |
|  | (“Cardiac Glycoside” AND Cancer [Title, Abstract & Keywords]) | 403 |
|  | (“Na+ K+ ATPase” AND Cancer [Title, Abstract & Keywords]) | 461 |
| Total | | 2880 |
|  | | |
|  | | |
| Total Literature Search | | 8946 |
