# Supplementary material for: Cardiac glycosides use and the risk and mortality of cancer; systematic review and meta-analysis of observational studies
Source: PLoS One. 2017 Jun 7;12(6):e0178611. doi: 10.1371/journal.pone.0178611 (PMC5462396; doi:10.1371/journal.pone.0178611)
Supplement: S2 Table — (DOCX) [file pone.0178611.s002.docx]

Supplementary Table 2.
Methodological quality of the included studies, based on the NOS for assessing the quality of epidemiological studies.

1. Case Control Studies (n=14)

| Study | **Selection** | | | | **Comparability** | **Exposure** | | | Total Score |
| --- | --- | --- | --- | --- | --- | --- | --- | --- | --- |
|  | Adequate definition of cases^1^ | Representativeness of cases | Selection of controls | Definition of controls | Control for  2 important factors^2,3^ | Ascertainment of exposure | Same method of ascertainment for cases and control | Non-response rate^4^ |  |
| Aromaa  1976 | * | * | - | * | ** | * | * | * | 8 |
| Casagrande 1988 | * | * | * | * | * | - | * | - | 6 |
| Lenfant-Pejovic 1990 | * | * | - | * | * | - | * | - | 5 |
| Bernstein 1992 | * | * | * | * | * | - | * | * | 7 |
| Friedman 1998 | * | * | * | * | * | - | * | * | 7 |
| Ewertz 2001 | * | * | * | * | * | - | * | - | 6 |
| Ahern 2008 | * | * | * | * | ** | * | * | * | 9 |
| Boursi 2014 | * | * | * | * | ** | * | * | * | 9 |
| Couraud ^35^ 2014 | * | * | - | * | ** | * | * | * | 8 |
| Couraud ^34^ 2014 | * | * | - | * | ** | * | * | * | 8 |
| Wright 2014 | * | * | * | * | ** | - | * | * | 8 |
| Kaapu 2015 | * | * | * | * | ** | * | * | * | 9 |
| Boursi 2016 | * | * | * | * | ** | * | * | * | 9 |
| Seliger 2016 | * | * | - | * | ** | * | * | * | 8 |

1. Cohort Studies (n=15)

| Study | **Selection** | | | | **Comparability** | **Exposure** | | | Total Score |
| --- | --- | --- | --- | --- | --- | --- | --- | --- | --- |
|  | Representativeness  of the exposed  cohort | Selection of the non-exposed cohort | Ascertainment of exposure^5^ | Outcome was not present at start of study^6^ | Control for  2 important factors^2,3^ | Assessment of outcome | Follow-up long enough | Adequacy of  follow-up of  cohort^7^ |  |
| Danielson 1982 | * | - | * | - | * | * | - | * | 5 |
| Friedman 1984 | * | * | * | * | * | * | * | - | 7 |
| Friedman 1989 | * | * | * | * | * | * | * | - | 7 |
| Haux 2001 | * | - | * | * | * | * | * | - | 6 |
| Biggar 2011 | * | * | * | - | * | * | * | * | 7 |
| Platz 2011 | * | * | - | * | ** | * | * | * | 8 |
| Biggar 2012 | * | * | * | - | ** | * | * | * | 8 |
| Hartz 2013 | * | * | - | * | * | - | * | * | 6 |
| Ahern 2014 | - | * | - | * | ** | * | * | * | 6 |
| Flahavan 2013 | * | * | * | * | ** | * | * | * | 9 |
| Karasneh ^50^ 2015 | * | * | * | - | ** | * | * | * | 8 |
| Karasneh ^51^ 2015 | * | * | * | - | ** | * | * | * | 8 |
| Karasneh 2016 | * | * | * | - | ** | * | * | * | 8 |
| Kaapu 2016 | * | * | * | - | ** | * | * | * | 8 |
| Vogel 2016 | * | * | * | - | * | * | * | * | 7 |

^1^ If the cases data was obtained from records with mentioning the process to extract information, or reference to primary record, a point was assigned.
^2^ If adjusted for age, a point was assigned.
^3^ If adjusted for drugs (e.g. anti-hypertensives, anti-diabetics etc) or any other additional factors, a point was assigned.
^4^ If information were obtained through national registries or hospital records, a point was assigned.
^4^ If information were obtained from patients and the difference in non-response rate between groups was 20% or less a point was assigned.
^5^ If the exposure data was obtained from prescription database or medical record, a point was assigned.
^6^ If the study design is prospective study, a point was assigned.
^7^ If the completeness of follow-up was 80% or more, a point was assigned.
