# Supplementary material for: Cardiac glycosides use and the risk and mortality of cancer; systematic review and meta-analysis of observational studies
Source: PLoS One. 2017 Jun 7;12(6):e0178611. doi: 10.1371/journal.pone.0178611 (PMC5462396; doi:10.1371/journal.pone.0178611)
Supplement: S1 File — (DOCX) [file pone.0178611.s003.docx]

**Supplementary file 1. Excluded studies with reasons:**

Twenty-Three Excluded Studies:

Five articles were excluded as no usable data were reported. [1–5]

The exposure of interest was not digoxin use in 7 studies.[6–12]

Five literature reviews without original data. [13–17]

Two studies whose participants were overlapped in other studies were excluded. [18,19]

Four conference abstracts were excluded as 1 reported incomplete data and 3 were published later as journal articles. [20–23]

1. Armstrong B, Stevens N, Doll R. RETROSPECTIVE STUDY OF THE ASSOCIATION BETWEEN USE OF RAUWOLFIA DERIVATIVES AND BREAST CANCER IN ENGLISH WOMEN. Lancet. Oxford University, United Kingdom; 1974;304: 672–675. doi:10.1016/S0140-6736(74)93258-9

2. LeWinn EB, Stenkvist B, Bengtsson E, Eriksson O, Holmquist J, Nordin B, et al. Cardiac glycosides and breast cancer. Lancet (London, England). ENGLAND; 1979. p. 563.

3. Stenkvist B, Bengtsson E, Dahlqvist B, Eriksson O, Jarkrans T, Nordin B. Cardiac glycosides and breast cancer, revisited. The New England journal of medicine. UNITED STATES; 1982. p. 484.

4. Stenkvist B. Is digitalis a therapy for breast carcinoma? Oncol Rep. 1999;6: 493–496.

5. Woopen H, Richter R, Chekerov R, Siepmann T, Ismaeel F, Sehouli J. The influence of comorbidity and comedication on grade III/IV toxicity and prior discontinuation of chemotherapy in recurrent ovarian cancer patients: An individual participant data meta-analysis of the North-Eastern German Society of Gynecological Oncolo. Gynecol Oncol. 2015;138: 735–740. doi:10.1016/j.ygyno.2015.07.007

6. Tenenbaum A, Grossman E, Fisman EZ, Adler Y, Boyko V, Jonas M, et al. Long-term diuretic therapy in patients with coronary disease: Increased colon cancer-related mortality over a 5-year follow-up. J Hum Hypertens. Cardiac Rehabilitation Institute, Bezafibrate Infarction Prevention Coordinating Center, Chaim Sheba Medical Center, Tel-Hashomer, Israel; 2001;15: 373–379. doi:10.1038/sj.jhh.1001192

7. Tenenbaum A, Motro M, Jonas M, Fisman EZ, Grossman E, Boyko V, et al. Is diuretic therapy associated with an increased risk of colon cancer? Am J Med. Cardiac Rehabilitation Institute, Bezafibrate Infarction Prevention Coordinating Center, Chaim Sheba Medical Center, Tel-Hashomer, Israel; 2001;110: 143–145. doi:10.1016/S0002-9343(00)00674-4

8. Paganinihill A. Aspirin and colorectal cancer: The Leisure World Cohort revisited. Prev Med (Baltim). Univ. So. Calif., Sch. Med., Dept. Prevent. Med., 1721 Griffin Ave. 200, Los Angeles, CA 90031, United States; 1995;24: 113–115. doi:10.1006/pmed.1995.1020

9. Pahor M, Guralnik JM, Ferrucci L, Corti M-C, Salive ME, Cerhan JR, et al. Calcium-channel blockade and incidence of cancer in aged populations. Lancet. Dept. of Int. Med. and Geriatrics, Catholic University, Rome, Italy; 1996;348: 493–497. doi:10.1016/S0140-6736(96)04277-8

10. Friis S, Poulsen AH, Johnsen SP, McLaughlin JK, Fryzek JP, Dalton SO, et al. Cancer risk among statin users: A population-based cohort study. Int J Cancer. Institute of Cancer Epidemiology, Danish Cancer Society, Copenhagen, Denmark; 2005;114: 643–647. doi:10.1002/ijc.20758

11. Biggar RJ, Andersen EW, Wohlfahrt J, Melbye M. Spironolactone use and the risk of breast and gynecologic cancers. CANCER Epidemiol. 2013;37: 870–875. doi:10.1016/j.canep.2013.10.004

12. Pottegård A, Friis S, Hallas J. Cancer risk in long-term users of vitamin K antagonists: A population-based case-control study. Int J Cancer. Clinical Pharmacology, University of Southern Denmark, Institute of Public Health, JB Winsløwsvej 19, 2, 5000 Odense C, Denmark; 2013;132: 2606–2612. doi:10.1002/ijc.27905

13. LeWinn EB. Cardiac glycosides and breast cancer. Lancet (London, England). ENGLAND; 1979. pp. 1196–1197.

14. Khan MI, Chesney JA, Laber DA, Miller DM. Digitalis, A Targeted Therapy for Cancer? Am J Med Sci. 2009;337: 355–359.

15. Biggar RJ. Molecular Pathways: Digoxin Use and Estrogen-Sensitive Cancers-Risks and Possible Therapeutic Implications. Clin CANCER Res. 2012;18: 2133–2137. doi:10.1158/1078-0432.CCR-11-1389

16. Digoxin and breast cancer. Prescrire Int. France; 2013;22: 98.

17. Masood S. Is digoxin a breast cancer risk factor? Acute cardiac care. England; 2015. pp. 29–31. doi:10.3109/17482941.2015.1066823

18. Walsh PC. Re: A Novel Two-Stage, Transdisciplinary Study Identifies Digoxin as a Possible Drug for Prostate Cancer Treatment. J Urol. 2012;187: 143.

19. Biggar RJ, Andersen EW, Kroman N, Wohlfahrt J, Melbye M. Breast cancer in women using digoxin: tumor characteristics and relapse risk. BREAST CANCER Res. 2013;15. doi:10.1186/bcr3386

20. Platz E, Yegnasubramanian S, Liu J, Shim J, Chong C, Stampfer M, et al. Digoxin users have a lower risk of prostate cancer than non-users in a large prospective cohort study. CANCER Res. 2009;69.

21. Flahavan E, Bennett K, Sharp L, Barron TI. Digoxin exposure and prostate cancer mortality: A matched cohort study. J Clin Oncol. 2012;30.

22. Flahavan EM, Bennett K, Sharp L, Barron TI. A Matched Cohort Study Examining Digoxin Exposure and Prostate Cancer Mortality. Pharmacoepidemiol Drug Saf. 2012;21: 343–344.

23. Lip S, Carlin C, McCallum L, Touyz RH, Dominiczak AF, Padmanabhan S. LB01.03: INCIDENCE AND PROGNOSIS OF CANCER ASSOCIATED WITH DIGOXIN AND COMMON ANTIHYPERTENSIVE DRUGS. J Hypertens. England; 2015;33 Suppl 1: e45. doi:10.1097/01.hjh.0000467465.54504.ae
