# Supplementary material for: Cardiac glycosides use and the risk and mortality of cancer; systematic review and meta-analysis of observational studies
Source: PLoS One. 2017 Jun 7;12(6):e0178611. doi: 10.1371/journal.pone.0178611 (PMC5462396; doi:10.1371/journal.pone.0178611)
Supplement: S3 File — (DOCX) [file pone.0178611.s005.docx]

Supplementary File 3. Forest plots of prostate cancer analysis

Forest plot for association between CGs use and risk of prostate cancer


Forest plot for association between CGs use and risk of prostate cancer – Stratified by study design

Forest plot for association between digoxin use and risk of breast cancer – Stratified by duration of drug usage (3 years or more)

Forest plot for association between digoxin use and risk of prostate cancer – Stratified by duration of drug usage (5 years or more)

Forest plot for association between digoxin use and risk of prostate cancer (Gleason Score 7 or More)

Forest plot for association between digoxin use and risk of prostate cancer (Advanced Stages) [advanced stage (≥T3b, N+ or M+ at diagnosis]:
