# Supplementary material for: Cardiac glycosides use and the risk and mortality of cancer; systematic review and meta-analysis of observational studies
Source: PLoS One. 2017 Jun 7;12(6):e0178611. doi: 10.1371/journal.pone.0178611 (PMC5462396; doi:10.1371/journal.pone.0178611)
Supplement: S4 File — (DOCX) [file pone.0178611.s006.docx]

Supplementary File 4. Forest plots of cancers colorectal, glioblastoma, male breast and lung

Forest plot for the association between CGs use and the risk of colorectal cancer


Forest plot for association between CGs use and the risk of colorectal cancer – Stratified by study design

Forest plot for the association between CGs use and the risk of Lung cancer

Forest plot for association between CGs use and risk of male breast cancer

Forest plot for association between CGs use and risk of glioblastoma
